# Nuciferine induces autophagy to relieve vascular cell adhesion molecule 1 activation via repressing the Akt/mTOR/AP1 signal pathway in the vascular endothelium

**DOI:** 10.3389/fphar.2023.1264324

**Published:** 2023-09-28

**Authors:** Haibin Wei, Yujie Yin, Wenwen Yang, Jinyan Zhu, Lin Chen, Rui Guo, Zhen Yang, Songtao Li

**Affiliations:** ^1^ School of Public Health, Zhejiang Chinese Medical University, Hangzhou, China; ^2^ Department of Biobank, Zhejiang Cancer Hospital, Hangzhou, Zhejiang, China; ^3^ School of Life Science, Zhejiang Chinese Medical University, Hangzhou, Zhejiang, China; ^4^ Department of Clinical Nutrition, Affiliated Zhejiang Hospital, School of Medicine, Zhejiang University, Hangzhou, Zhejiang, China

**Keywords:** nuciferine, VCAM1, autophagy, AP1, AKT/mTOR, vascular endothelium

## Abstract

Pro-inflammatory factor-associated vascular cell adhesion molecule 1 (VCAM1) activation initiates cardiovascular events. This study aimed to explore the protective role of nuciferine on TNFα-induced VCAM1 activation. Nuciferine was administrated to both high-fat diet (HFD)-fed mice and the TNFα-exposed human vascular endothelial cell line. VCAM1 expression and further potential mechanism(s) were explored. Our data revealed that nuciferine intervention alleviated VCAM1 activation in response to both high-fat diet and TNFα exposure, and this protective effect was closely associated with autophagy activation since inhibiting autophagy by either genetic or pharmaceutical approaches blocked the beneficial role of nuciferine. Mechanistical studies revealed that Akt/mTOR inhibition, rather than AMPK, SIRT1, and p38 signal pathways, contributed to nuciferine-activated autophagy, which further ameliorated TNFα-induced VCAM1 via repressing AP1 activation, independent of transcriptional regulation by IRF1, p65, SP1, and GATA6. Collectively, our data uncovered a novel biological function for nuciferine in protecting VCAM1 activation, implying its potential application in improving cardiovascular events.

## Introduction

Vascular cell adhesion molecule 1 (VCAM1) is a transmembrane protein and consists of an intracellular (C-terminal) and extracellular (N-terminal) portion. The extracellular portion contains several immunoglobulin (Ig)-like domains, which are responsible for tight adhesion with immune cells through the interaction with α4β1, α4β7, α9β1, and αDβ2 integrins (Elices et al., 1990; Ulyanova et al., 2005). VCAM1 rarely expresses in endothelial cells under physiological conditions. However, it could be quickly induced/activated directly or indirectly by various stimuli, such as pro-inflammatory factors [e.g., tumor necrosis factor alpha (TNFα), interleukin-1 beta (IL-1β), and interferon-gamma (IFN-γ)] (Carlos et al., 1990; Neish et al., 1992; Paleolog et al., 1992), oxidative stresses [e.g., reactive oxygen species (ROS) and oxidative modification of low-density lipoprotein (ox-LDL)] (Yoshida et al., 2000; Lee et al., 2007; Zhu et al., 2008), and infections (e.g., coronavirus disease 2019, human immunodeficiency virus, and human cytomegalovirus) (Liu et al., 2005; Zhao et al., 2018; Robles et al., 2022). Endothelial dysfunction and ongoing cardiovascular inflammation are caused by immune cells penetrating the arterial wall as a result of activated VCAM1 on endothelial cells, which attracts leukocytes, monocytes, and neutrophils (Goswami et al., 2021). Unsettled cardiovascular inflammation further aggravates VCAM1 activation, which leads to the initiation of cardiovascular events. Accumulated clinical and animal studies have shown that VCAM1 is activated in different cardiovascular events, such as atherosclerosis, stroke, and heart failure (Troncoso et al., 2021). Cardiovascular events are the leading causes of death globally (Khoury et al., 2021), but effective preventions or interventions are still limited (Lu and Thum, 2019). There is an urgent need for studies that try to identify novel therapeutic approaches. As a reversible process, VCAM1 activation is recognized as an ideal therapeutic target for the prevention/intervention of cardiovascular events at an early stage or other endothelial dysfunction-related diseases (Carter et al., 2002; Park et al., 2013; Furuta et al., 2021).

*Nelumbo nucifera* Gaertn (also known as lotus) is an edible medicinal plant recorded by both Ayurveda and Chinese traditional medicine (Wang et al., 2015). Lotus is constituted mainly by lotus leaves, flower, rhizomes, and seeds, among which lotus leaf is commonly consumed as tea and medicine. Nuciferine (C19H21NO2) is the main bioactive component obtained from lotus leaves. As an aporphine alkaloid, nuciferine has been reported with extensive pharmacological functions, including anti-inflammatory, anti-oxidant, anti-obesity, anti-hepatic steatosis, and anti-tumor properties (Wu et al., 2017; Bai et al., 2022; Du et al., 2022). Since inflammation, oxidative stress, and obesity are risk factors for VCAM1 activation and further cardiovascular events (Ross, 1999; Davis et al., 2011; Yannakoulia and Panagiotakos, 2021), we proposed that nuciferine protects pro-inflammatory factor-induced activation of VCAM1. To the best of our knowledge, limited study has been conducted to investigate the beneficial role of nuciferine against VCAM1 activation. In the present study, we reported for the first time that nuciferine intervention improved both high-fat diet (HFD) and TNFα-induced VCAM1 activation. Further mechanistic study revealed that nuciferine-stimulated autophagy via suppressing Akt/mTOR contributed to its preventive role against VCAM1 activation.

## Materials and methods

### Chemicals and reagents

Nuciferine (purity by HPLC ≥98%) was obtained from Chengdu Must Bio-Technology Co., Ltd. (Chengdu, China), and dissolved in dimethyl sulfoxide (DMSO) in a 10 mmol/L stock. The chemical structure of nuciferine was drawn using ChemDraw 21.0.0 software (PerkinElmer, Waltham, MA). Rapamycin, SB202190, chloroquine (CQ), asiatic acid, and MK2206 were purchased from Selleck (Shanghai, China). CQ was dissolved in sterilized water, while rapamycin, SB202190, asiatic acid, and MK2206 were dissolved in DMSO. Recombinant human TNFα was bought from R&D Systems (Minneapolis, MN) and dissolved in sterile phosphate-buffered saline (PBS) containing 0.5% bovine serum albumin (BSA) before use. Information about all antibodies is shown in Table 1.

**TABLE 1:** Antibodies information.

Antibody	Source	Cat. no.	Dilution	Application
VCAM1	Abcam	ab134047	1–1,000	WB
			1–200	IHC IF
c-Fos	Abcam	ab208942	1–1,000	WB
GAPDH	Boster	BM1632	1:10,000	WB
p-p38 (Thr180/Tyr182)	CST	4511S	1–1,000	WB
Total p38	CST	9212S	1–1,000	WB
P-AKT (Ser473)	CST	4060S	1–1,000	WB
Total AKT	CST	9272S	1–1,000	WB
p-p70S6K (Thr389)	CST	9234S	1–1,000	WB
Total p70S6K	CST	2708S	1–1,000	WB
ATG5	CST	12994S	1–1,000	WB
ATG12	CST	4180S	1–1,000	WB
Beclinl	CST	3495S	1–1,000	WB
c-Jun	CST	9165S	1–1,000	WB
GATA6	CST	5851S	1–1,000	WB
SP1	CST	9389S	1–1,000	WB
NFKB (p65)	CST	8242S	1–1,000	WB
LC3	Sigma	L7543	1–3,000	WB
Goat anti-rabbit IgG	Boster	BA1054	1:10,000	WB
Goat anti-mouse IgG	Boster	BA1050	1:10,000	WB

Cat. no., catalog number; CST, cell signaling technology; WB, Western blot; IHC, immunohistochemistry; IF, immunofluorescence.

### Animal handling

C57BL/6J male mice (6 weeks old) were obtained from Shanghai SLAC Laboratory Animal Co., Ltd. (Shanghai, China), and kept in a specific pathogen-free (SPF) laboratory with free access to food and water. All animal experiments were approved by the Laboratory Animal Management and Ethics Committee of Zhejiang Chinese Medical University (Approval No. IACUC-20210423-08). After 1 week of acclimatization, all mice were randomly assigned into a control group (*n* = 5), model group (*n* = 5), and nuciferine group (*n* = 5). The mice in the control group were fed a normal-fat diet (NFD; TP26352, TROPHIC Animal Feed High- Tech Co., Ltd., Nantong, China). The mice in the model group were fed an HFD (TP26304, TROPHIC Animal Feed High- Tech Co., Ltd.). Mice in the NUC group were fed an HFD containing 0.06% (w/w of the HFD) nuciferine supplement. The dosage of nuciferine referred to the low dose that Zhang *et al.* reported (Zhang et al., 2018). Caloric compositions of each diet are listed in Table 2. After 16 weeks of feeding, all mice were anesthetized and euthanized, and the plasma and aortic arch tissues were collected and stored in −80°C for further experiments.

**TABLE 2:** Caloric compositions of each diet.

Constituents	Normal-fat diet	High-fat diet
Heat density, kilocalorie/g	3.5	4.5
Protein	14%	14%
Carbohydrate	76%	44%
Fat	10%	42%
Total	100%	100%

### Enzyme-linked immunosorbent assay

VCAM1 content in mice plasma was determined using a commercial enzyme-linked immunosorbent assay (ELISA) kit obtained from Cusabio (CSB-E04754m, Wuhan, China). The assay was carried out according to the manufacturer’s instructions. In brief, plasma and standard were first diluted with sample dilution and then added to the coated assay plate, followed by incubation at 37°C. Two hours later, after the liquid was removed, the assay plate was incubated with a biotin antibody at 37°C for 1 h and washed three times. HRP-avidin was then added, and the plate was incubated again at 37°C. One hour later, after washing five times, a TMB substrate was added and incubated at 37°C for 20 min. At last, a stop solution was added to stop the reaction, and the optical density (OD) at 450 nm and 570 nm was determined using a FLUOstar Omega microplate reader (BMG LABTECH, Ortenberg, Germany). The final data were calculated by subtracting the OD at 570 nm from the OD at 450 nm.

### Immunohistochemistry

Aortic arch tissues were fixed in 5% paraformaldehyde, embedded in an optimal cutting temperature (OCT) compound (4583, SAKURA, Torrance, CA), and frozen in liquid nitrogen. Frozen sections of 8 μm were cut using a frozen section machine (HM525, Thermo Scientific, MA) and then incubated with 10% goat serum. After that, the sections were incubated with a primary antibody against VCAM1 (1:50) at 4°C overnight, washed with PBS, and then, incubated with the Alexa Fluor^®^ 488-conjugated Goat Anti-Rabbit IgG (H + L) secondary antibody (1:150, DW-GAR4881, Dawen Biotec, Hangzhou, China). The primary and secondary antibodies were diluted in 10% goat serum. Eventually, nuclei were stained with 2-(4-amidinophenyl)-6-indolecarbamidine dihydrochloride (DAPI) staining solution (C1005, Beyotime, Shanghai, China) for 10 min. Images were captured using a digital pathology scanner (Olympus, VS120-S6-W, Tokyo, Japan).

### Cell culture

The human vascular endothelial cell line, EA.hy926, was obtained from the American Type Culture Collection (ATCC) (Manassas, VA) and cultured with Dulbecco’s modified Eagle’s medium (DMEM) (HyClone, Logan, UT) supplemented with 10% fetal bovine serum (FBS) (CellMax, Beijing, China). The cell line was routinely maintained on the 100-mm tissue culture dish in the 37°C incubator with humidified 95% air and 5% CO_2_.

### Cell viability assay

4 × 10^3^ cells/well were seeded onto a 96-well tissue culture plate; 16 h later, the culture medium was replaced with a fresh medium with nuciferine (0, 1, 2.5, 5, 10, and 20 μmol/L) for another 24 h. Cell viability was determined using a cell-counting kit 8 (CCK8) (Bimake, Houston, TX) as we previously described (Zhou et al., 2020).

### RNA interfering

Genes targeting small interfering RNA (siRNA) and scrambled siRNA (as the negative control) were synthesized by GenePharma (Shanghai, China). 10 nmol/L siRNA was delivered to cells with RNAiMAX as we previously described (Zhou et al., 2020). Human ATG5, c-Jun, and c-Fos siRNA-targeting sequences (5′-3′) are CCT​TTG​GCC​TAA​GAA​GAA​A (ATG5), GUC​AUG​AAC​CAC​GUU​AAC​A (c-Jun), and GCA​AUA​GUG​UGU​UCU​GAU​U (c-Fos).

### Western blot assay

Cells were harvested by scratching and resuspended in pre-cold PBS. RIPA buffer (Boster, Wuhan, China) or a nuclear and cytoplasmic protein extraction kit (CWBIO, Beijing, China) was utilized to extract the total or nuclear protein, respectively. Western blot assay was carried out following a previous protocol (Li et al., 2013). Final data were visualized under a ChemiScope Series machine from Clinx Science Instruments Co., Ltd. (3300 Mini) (Shanghai, China). The internal control for total protein (GAPDH) or nuclear protein (Histone H3) was employed. Relative protein expressions in all blots were quantized using ImageJ (NIH Image, Bethesda, MD). Representative blots from at least three repeats were shown.

### RNA extraction and quantitative polymerase chain reaction

Total RNA was extracted using a total RNA extractor (Sangon Biotech, Shanghai, China). Reverse transcription was then carried out to generate cDNA from RNA using HiScript III All-in-one RT SuperMix (Vazyme, Nanjing, China). SYBR green was utilized to determine the relative gene expression level with the 2^−ΔΔCT^ method. *ACTB* was set as the reference gene. Sequences for gene-specific primers are listed in Table 3.

**TABLE 3:** Sequences of gene-specific primers.

Gene symbol	Forward (5′-3′)	Reverse (3′-5′)
*ACTB*	ACTATCGGCAATGAGCG	GAGCCAGGGCAGTAATCT
*VCAM1*	TTC​CCT​AGA​GAT​CCA​GAA​ATC​GAG	CTT​GCA​GCT​TAC​AGT​GAC​AGA​GC
*c-JUN*	GGA​TCA​AGG​CGG​AGA​GGA​AG	CAC​CTG​TTC​CCT​GAG​CAT​GT
*c-FOS*	CTG​TCA​ACG​CGC​AGG​ACT​TC	TCA​TGG​TCT​TCA​CAA​CGC​CA

### Analysis of the autophagic flux

Lentivirus encoding GFP-LC3 (GeneChem, Shanghai, China) was delivered to cells to visualize autophagic flux. The autophagic flux was determined using a laser scanning confocal microscope (LSM 880, ZEISS, Jena, Germany). GFP-positive autophagic puncta were counted and quantified in each group (at least 20 cells were included per group) according to a previous study.

### Statistical analysis

All data were expressed as the mean ± SD. Statistical analysis was performed using unpaired Student’s *t*-test with GraphPad Prism 8.02 software (GraphPad Software, San Diego, CA). When *p* < 0.05, differences between the compared groups were considered to be significant.

## Results

### Nuciferine protects TNFα-induced VCAM1 in the vascular endothelium

**FIGURE 1:**
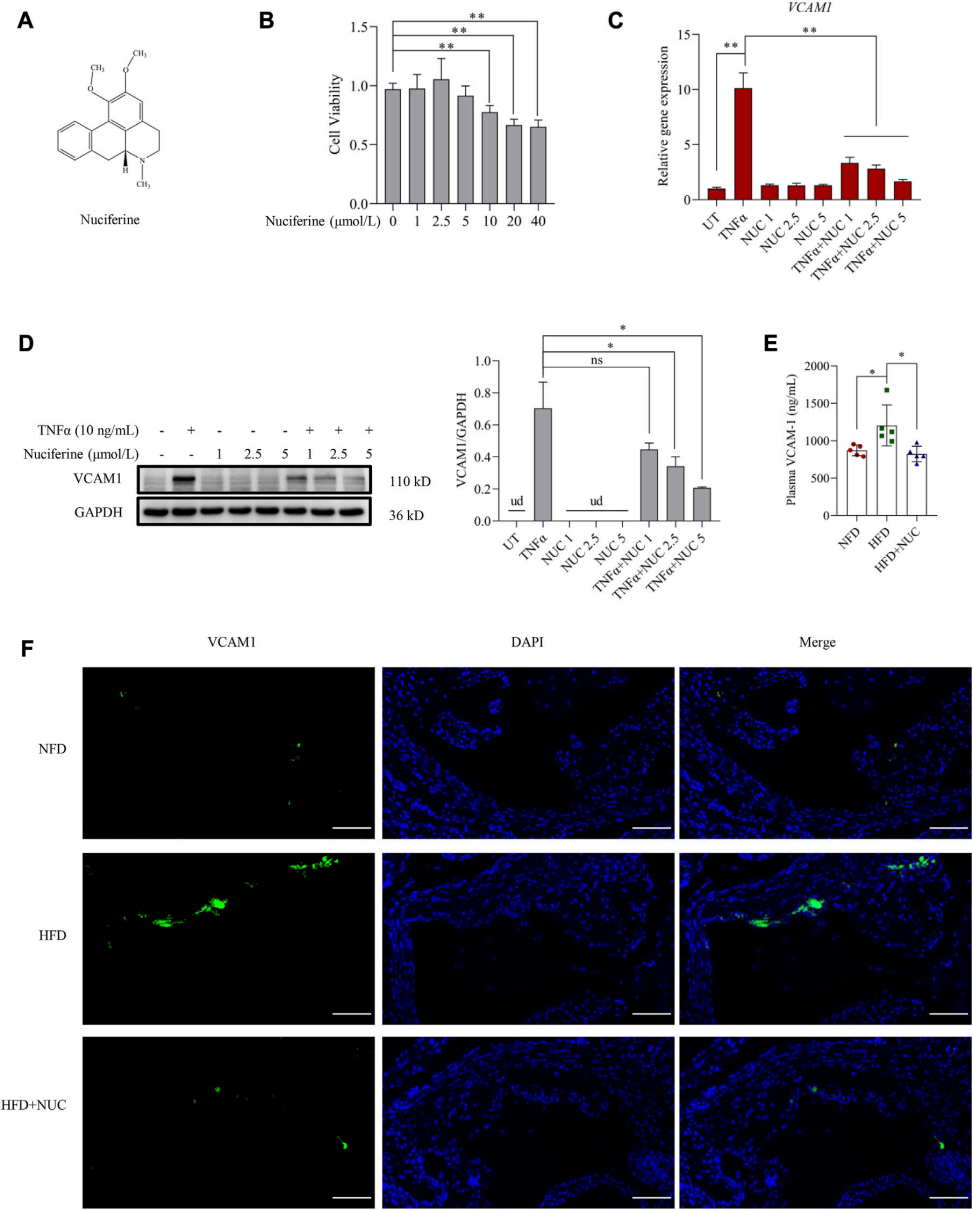
Nuciferine represses VCAM1 activation both *in vitro* and *in vivo*. **(A)** Chemical structure of nuciferine. **(B)** Cell viability test in EA.hy926 cells under 24 h treatment of nuciferine (0, 1, 2.5, 5, 10, 20, and 40 μmol/L). Relative mRNA level of VCAM1 verified by qPCR **(C)**, protein level of VCAM1 verified by Western blot assay **(D)** (left panel), and the relative quantification data **(D)** (right panel), while EA.hy926 cells were pretreated with nuciferine (0, 1, 2.5, and 5 μmol/L) for 2 h, followed by 12 h incubation with TNFα (10 ng/mL) or vehicle. **(E)** Plasma VCAM1 level examined by the ELISA method from the indicated mice. **(F)** VCAM1 immunofluorescence staining of arterial endothelia tissues from the indicated mice; nuclei were visualized by DAPI staining (blue), and VCAM1 was visualized by an FITC-conjugated secondary antibody (green); the final data were captured under a fluorescence digital pathology scanner (magnification ×200; scale bar, 100 μm). All values are denoted as the means ± SD from five animal samples or at least three independent batches of cells. Each group contains the same amount of solvent. ud, undetectable. ∗*p* < 0.05 and ∗∗*p* < 0.01 indicate statistically significant differences.

The chemical structure of nuciferine is shown in Figure 1A. As an aporphine alkaloid, nuciferine might be toxic when consumed beyond a certain concentration; therefore, cell viability was first assessed in the presence of nuciferine (0, 1, 2.5, 5, 10, 20, and 40 μmol/L) in endothelial cells. No significant cytotoxicity was observed when the dose of nuciferine was lower than 5 μmol/L (Figure 1B). Then, a commonly employed cellular model for VCAM1 activation was established by TNFα (10 ng/mL, 12 h) treatment in the EA.hy926 human vascular endothelial cell line. As shown in Figures 1C, D, TNFα treatment robustly stimulated VCAM1 expression at both transcriptional and protein levels. To investigate the protective role of nuciferine against VCAM1 activation, cells were pretreated with incremental doses of nuciferine (0, 1, 2.5, and 5 μmol/L) for 1 h before TNFα exposure. Our data clearly showed that nuciferine (the optimal dose is 5 μmol/L) significantly reversed TNFα-induced VCAM1 activation (Figures 1C, D). To consolidate our findings, we established an obesity-associated VCAM1 elevation model via feeding mice with an HFD, and changes in plasma TC, plasma TG, plasma HDL-C, plasma LDL-C, and mouse body weight confirmed the success of the animal model (Supplementary Figures S4A–E). As shown in Figures 1E, F, the HFD induced a significant increase in VCAM1 in both the arterial endothelium and blood while nuciferine administration rescued HFD-increased VCAM1 (Figures 1E, F). These data clearly indicated the protective ability of nuciferine against VCAM1 activation.

### Autophagy regulates VCAM1 activation in challenge to TNFα

**FIGURE 2:**
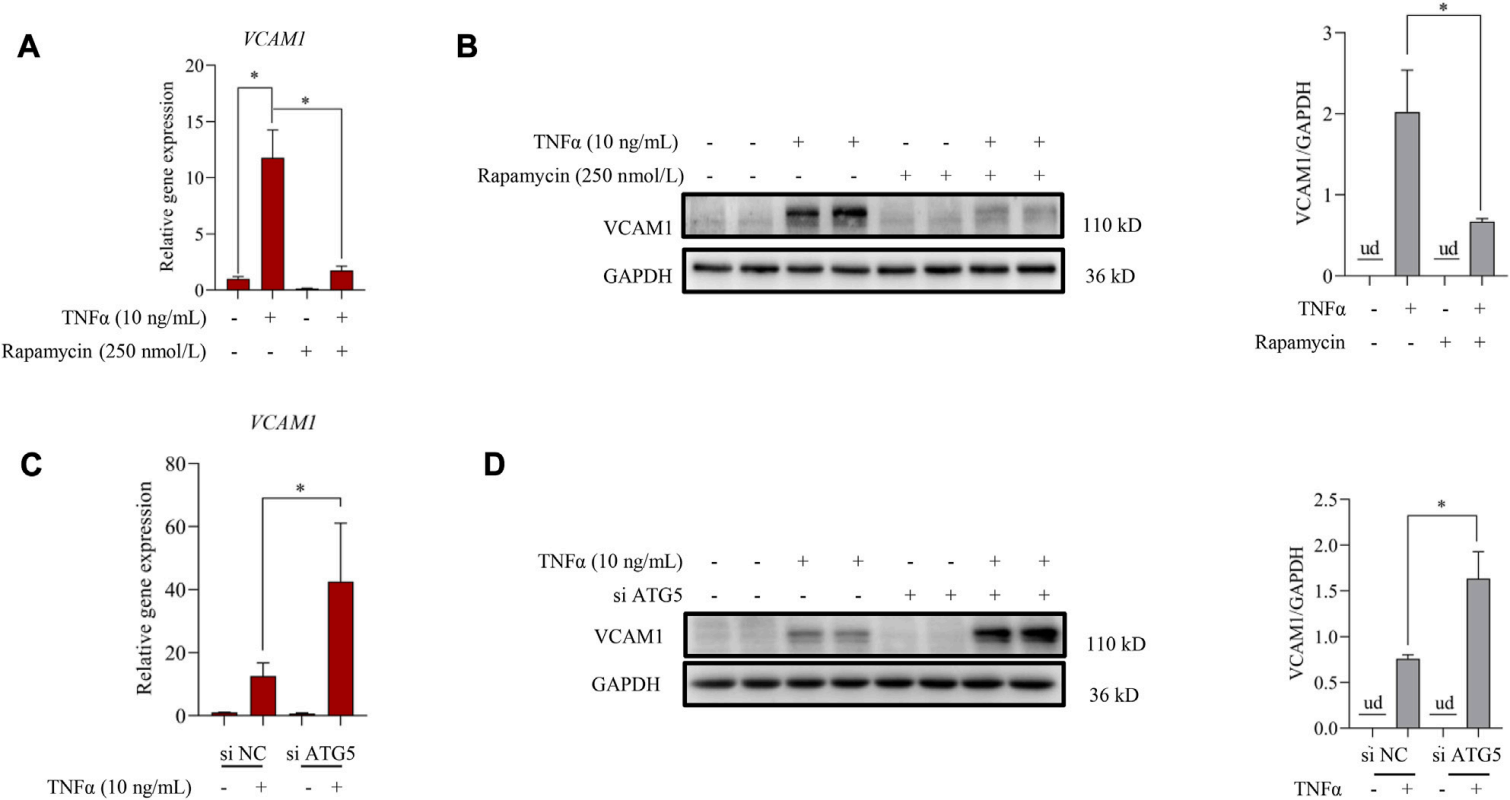
Autophagy contributes to TNFα-induced VCAM1 activation. **(A)** Relative mRNA level of VCAM1. **(B)** VCAM1 protein expression and the quantification data; EA.hy926 cells were pretreated with rapamycin for 2 h, followed by 12 h TNFα stimulation. **(C)** Relative VCAM1 gene expression level, **(D)** VCAM1 protein expression level and the quantification data; EA.hy926 cells were treated with si ATG5 or scrambled siRNA (si NC) for 24 h and then incubated with TNFα or vehicle for another 12 h. All values are denoted as the means ± SD from at least three independent tests. Each group contains the same amount of solvent. ud, undetectable. ∗*p* < 0.05 and ∗∗*p* < 0.01 indicate statistically significant differences.

Autophagy induction provided an effective strategy to improve VCAM1 activation (Wei et al., 2020). In this study, we observed that pre-incubation of human vascular endothelial cells with rapamycin, a commonly used autophagy agonist, significantly reversed TNFα-induced VCAM1 activation at both mRNA (Figure 2A) and protein levels (Figure 2B). Moreover, we established an instantaneous autophagy suppression model by transfecting endothelial cells with specific interfering RNA targeting on ATG5 (siATG5). The data revealed that ATG5 was effectively silenced by siRNA (Supplementary Figure S1), while autophagy suppression aggravated TNFα-induced VCAM1 activation at both mRNA and protein levels (Figures 2C, D). These results collectively indicated that autophagy negatively regulates VCAM1 expression in human vascular endothelial cells when challenged to TNFα exposure.

### Autophagy stimulation contributes to nuciferine-alleviated VCAM1 activation

**FIGURE 3:**
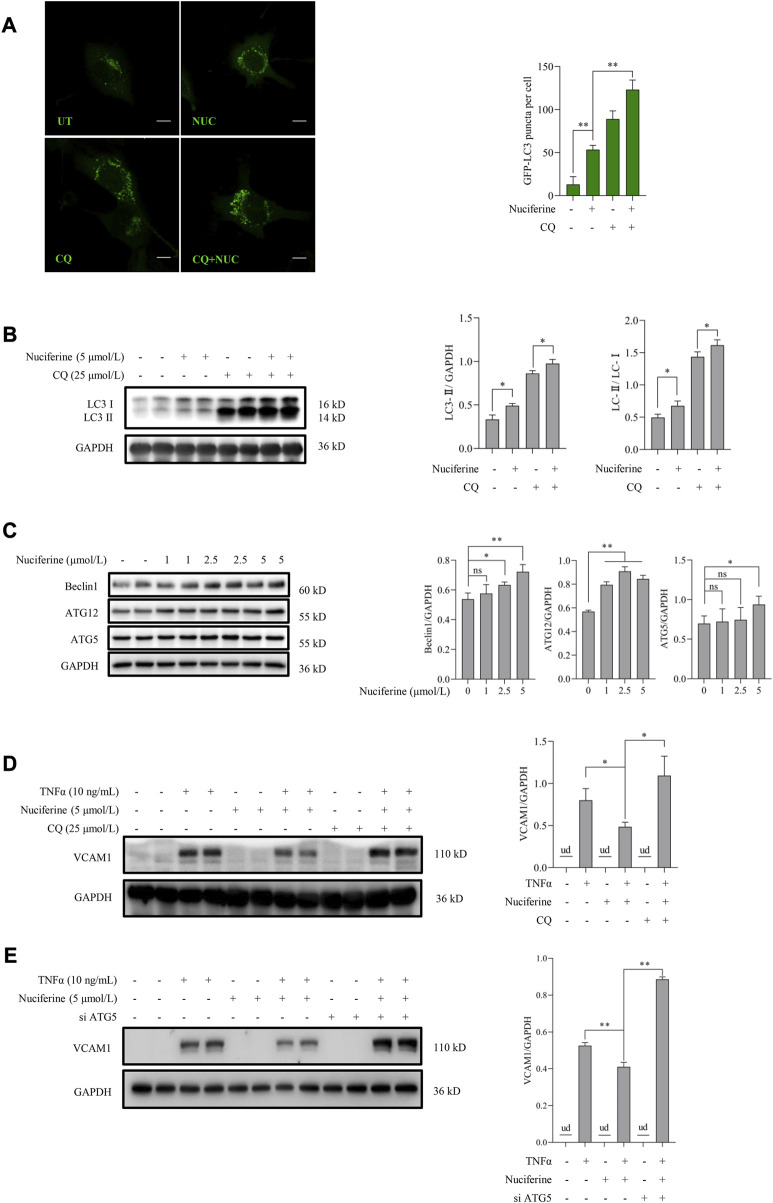
Nuciferine stimulates autophagy to protect against TNFα-induced VCAM1 activation. **(A)** GFP-LC3 puncta detected by confocal and bar graphs of the quantification data. The quantification data were generated from at least 20 different cells in each group. Scale bar, 20 μm. **(B)** LC3 Ⅰ/Ⅱ protein level and the quantification data. Cells in **(A,B)** were treated with CQ for 2 h ahead of nuciferine treatment. **(C)** Immunoblots of autophagy initiation-related proteins (Beclin1, ATG12, and ATG5) and the quantification data in EA.hy926 cells treated with nuciferine (0, 1, 2.5, and 5 μmol/L) for 12 h. **(D,E)** Protein level of VCAM1, as well as the quantification data in EA.hy926 cells, pretreated with CQ for 2 h **(D)** or siRNAs for 24 h **(E)** and then nuciferine (5 μmol/L) for 2 h, followed by 12 h TNFα incubation. All values are denoted as the means ± SD from at least three independent tests. Each group contains the same amount of solvent. ud, undetectable. ∗*p* < 0.05 and ∗∗*p* < 0.01 indicate statistically significant differences. ns, no significant differences.

We further investigated whether autophagy stimulation contributes to nuciferine-improved VCAM1 activation. According to our knowledge, the regulation of nuciferine on autophagy in vascular endothelial cells remains unknown. Here, we observed for the first time that nuciferine enhanced autophagy in human vascular endothelial cells based on the following findings: 1) nuciferine incubation increased microtubule-associated protein 1 lightchain 3 (MAP1LC3/LC3) autophagic puncta in the GFP-LC3-lentivirus-infected vascular endothelial cells (Figure 3A); 2) nuciferine treatment enhanced the expression of LC3 Ⅱ (lipidated form of LC3, a widely used marker for autophagic activation (Figure 3B); 3) nuciferine intervention promoted autophagic flux (Figures 3A, B) since nuciferine stimulated both LC3 puncta and LC3 Ⅱ expression in the presence of CQ, a well-employed autophagy antagonist through inhibiting autophagosome degradation in lysosome; and 4) nuciferine promoted the expression of autophagy initiation-related proteins, including Beclin1, ATG5, and ATG12 (Figure 3C). These data implied that nuciferine is a potential phytogenic autophagy inducer in vascular endothelial cells. More importantly, we explored whether autophagy stimulation was involved in nuciferine-alleviated VCAM1 activation. Autophagy was chemically or genetically suppressed by CQ or siRNA knocking down ATG5, respectively. Our data showed that nuciferine failed to improve TNFα-induced VCAM1 activation after autophagy repression (Figures 3D, E), implying that nuciferine-stimulated autophagy contributed to its preventive benefit against VCAM1 activation.

### p38 MAPK is irrelevant to nuciferine-reduced VCAM1 via autophagy activation

**FIGURE 4:**
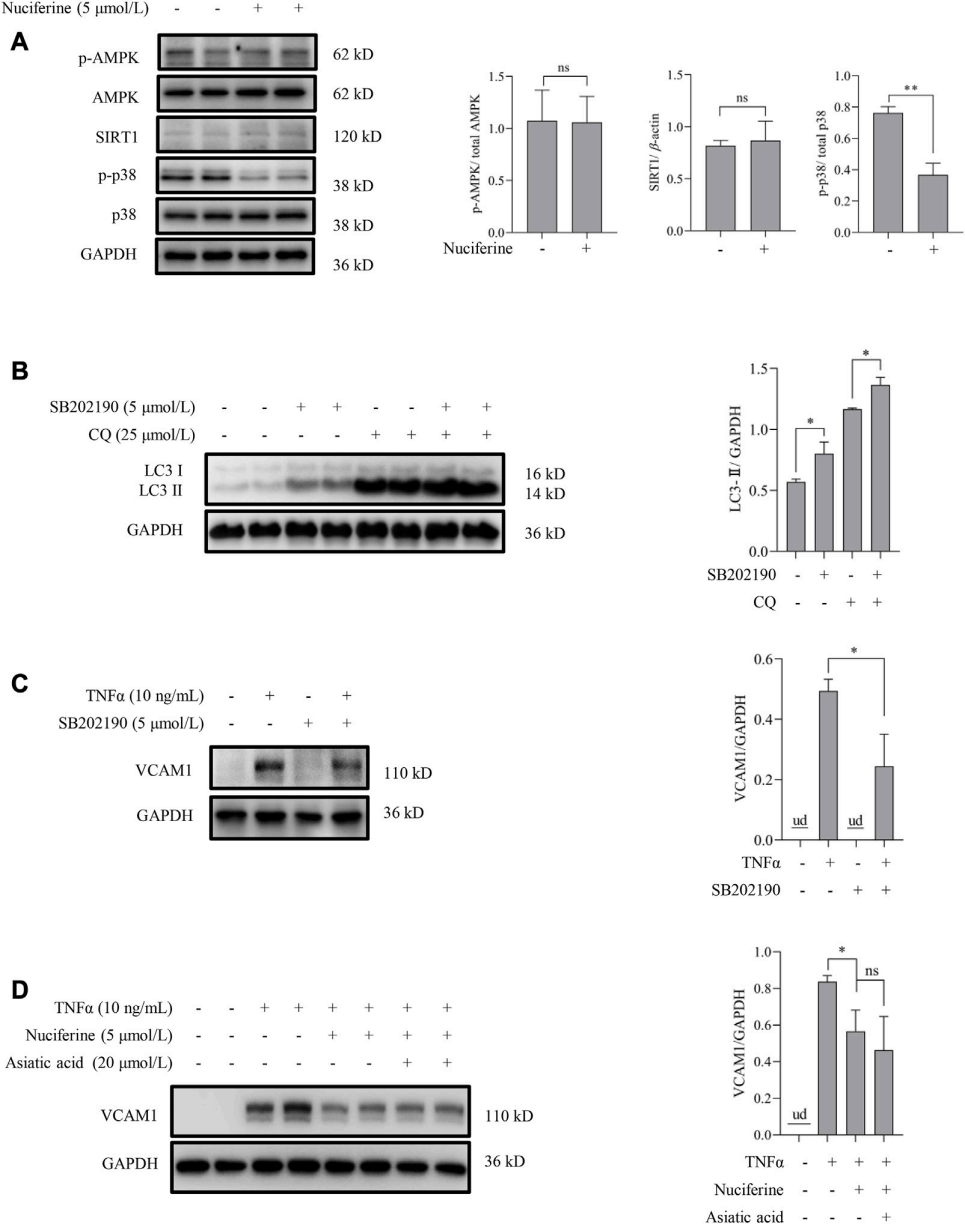
p38 MAPK is irrelevant to autophagy activation and VCAM1 reduction in response to nuciferine. **(A)** Upstream molecules for autophagy induction (p-AMPK, SIRT1, and p-p38) in the presence of nuciferine (6 h) detected by Western blot assay and the quantification data. **(B)** LC3 Ⅰ/Ⅱ protein level and the quantification data in EA.hy926 cells pretreated with CQ for 2 h, followed by SB202190 (5 μmol/L) treatment for 12 h. **(C)** Immunoblots of VCAM1 and the quantification data in cells pretreated with SB202190 (5 μmol/L) for 2 h, followed TNFα treatment for 12 h **(D)** VCAM1 protein expression and the quantification data in cells pretreated with nuciferine (5 μmol/L) and asiatic acid for 2 h, followed by TNFα treatment for 12 h. All values are denoted as the means ± SD from at least three independent tests. Each group contains the same amount of solvent. ud, undetectable. ∗*p* < 0.05 indicate statistically significant differences. ns, no significant differences.

We aimed to explore the upstream signaling pathways through which nuciferine stimulated autophagy to prevent VCAM1 activation. The involvement of several autophagy regulatory signaling pathways, including AMP-activated protein kinase (AMPK) (Kim et al., 2011; Zhou et al., 2019), silent information regulator sirtuin 1 (SIRT1) (Yuan et al., 2020; Lohanathan et al., 2022), and p38 mitogen-activated protein kinases (p38 MAPK) (Webber, 2010; Song et al., 2020), which have been reported to be modulated by nuciferine or its analog, was investigated in this study. Our data indicated that neither phosphorylated AMPK nor SIRT1 was stimulated by nuciferine incubation (Figure 4A), which preliminarily ruled out their participation in nuciferine-promoted autophagy. However, phosphorylated p38 (p-p38) was significantly reduced by nuciferine treatment when compared with that in the control group (Figure 4A). Correspondingly, p38 MAPK pathway inhibition by its special chemical antagonist SB202190 markedly promoted autophagic flux in vascular endothelial cells (Figure 4B). SB202190 treatment also partially protected the endothelial cells from TNFα-induced VCAM1 activation (Figure 4C). However, p38 MAPK induction by its pharmacological agonist asiatic acid could not block the protective role of nuciferine against TNFα-induced VCAM1 activation (Figure 4D). These data collaboratively indicated that p38 inhibition stimulated autophagy and improved TNFα-induced VCAM1 activation; however, such regulation was not the main mechanism of the beneficial effect of nuciferine on VCAM1 reduction.

### Akt pathway contributes to nuciferine-stimulated autophagy and -protected VCAM1 activation

**FIGURE 5:**
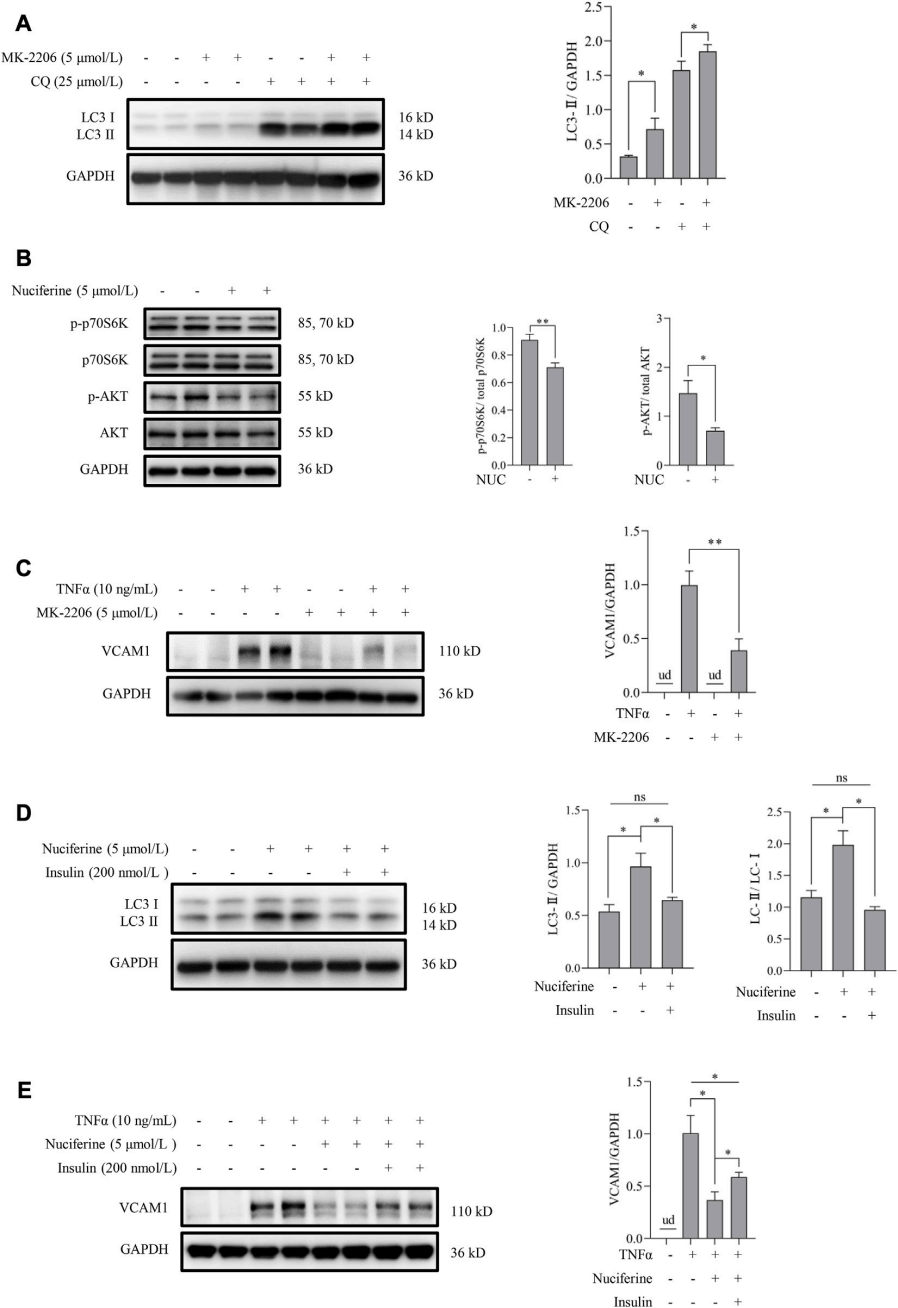
Nuciferine activates autophagy through Akt/mTOR inhibition to repress VCAM1 activation. **(A)** LC3 Ⅰ/Ⅱ protein level and the quantification data in cells pretreated with CQ for 2 h, followed by MK-2206 (5 μmol/L) treatment for 12 h. **(B)** Status of molecules in Akt/mTOR signaling (p-Akt, Akt, p-p70S6K, and p70S6K) under nuciferine treatment for 6 h and the quantification data. **(C)** VCAM1 protein expression and the quantification data in cells pretreated with MK-2206 (5 μmol/L) for 2 h, followed by TNFα treatment for 12 h. **(D)** LC3 Ⅰ/Ⅱ protein level and the quantification data in cells pretreated with insulin (200 nmol/L) for 2 h, followed by nuciferine treatment for 12 h. **(E)** VCAM1 protein expression and the quantification data in cells pretreated with nuciferine (5 μmol/L) and insulin (200 nmol/L) for 2 h, followed by TNFα treatment for 12 h. All values are denoted as the means ± SD from at least three independent tests. Each group contains the same amount of solvent. ud: undetectable. ∗*p* < 0.05 and ∗∗*p* < 0.01 indicate statistically significant differences. ns, no significant differences.

Akt inhibition has been reported to be associated with autophagy activation via regulating the mammalian target of rapamycin (mTOR) pathway (Takeuchi et al., 2005). In this study, we first tested the regulatory role of the Akt pathway on autophagy in human vascular endothelial cells. Our data showed that inhibiting Akt using a pan Akt antagonist MK-2206 significantly increased the autophagic flux (Figure 5A). Subsequently, the involvement of the Akt/mTOR pathway in nuciferine-stimulated autophagy and -protected VCAM1 activation was evaluated. Our data revealed that nuciferine treatment reduced the basal level of phosphorylated Akt (p-Akt on Ser473) and phosphorylated p70 S6 kinase (p-p70S6K on Thr389), a well-recognized target to reflect mTOR activity (Figure 5B). Similar with nuciferine treatment, Akt inhibition by MK-2206 also protected the endothelial cells from TNFα-induced VCAM1 activation (Figure 5C). More importantly, Akt activation, using its special physiological agonist insulin, strongly abolished nuciferine-induced autophagy activation (Figure 5D) and blocked the preventive role of nuciferine on VCAM1 activation (Figure 5E). These results indicated that Akt suppression contributed to nuciferine-stimulated autophagy and further protection on VCAM1 activation.

### Nuciferine alleviates TNFα-induced AP1 activation

**FIGURE 6:**
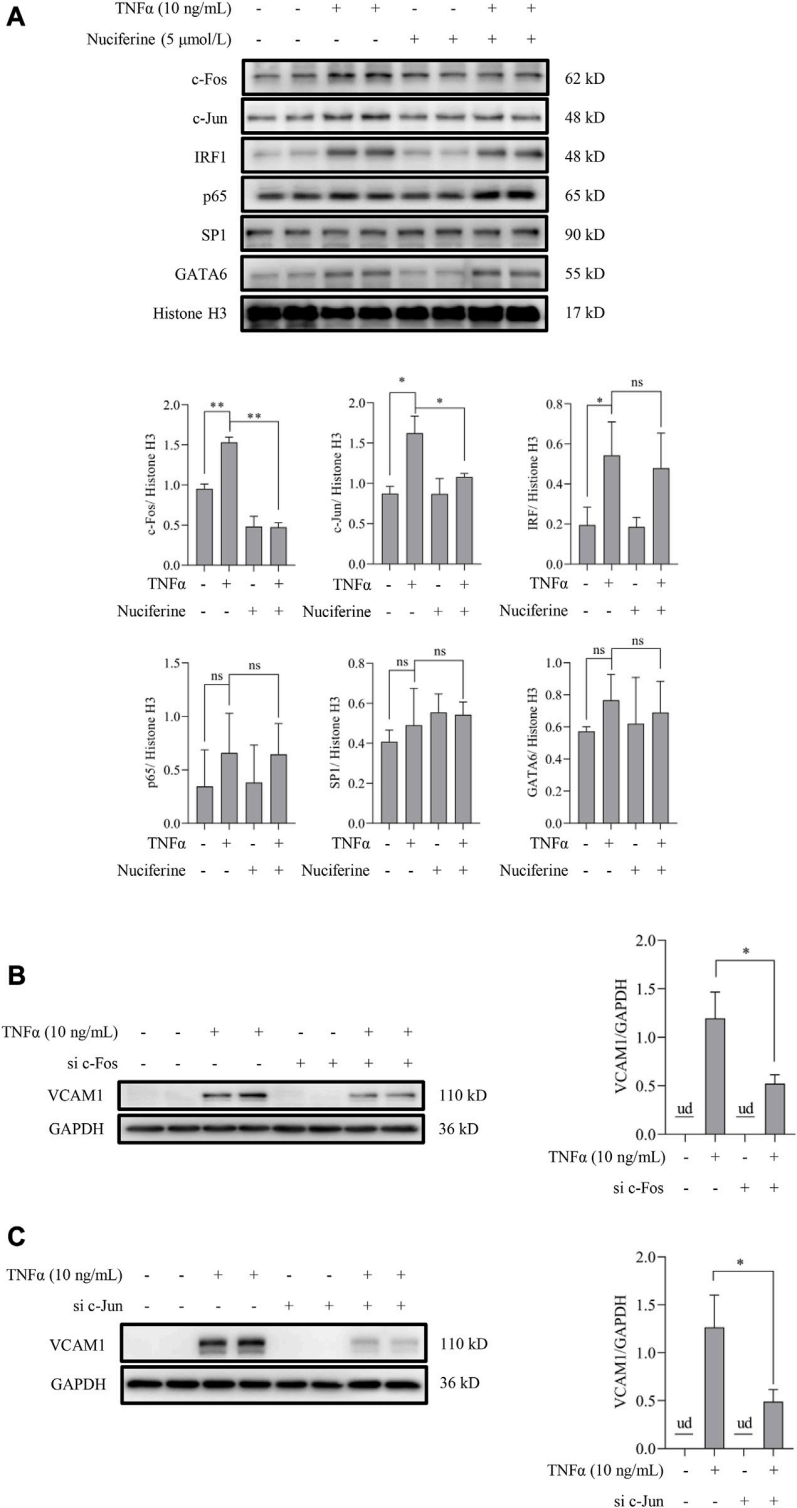
Nuciferine represses TNFα-induced nuclear translocation of AP1. **(A)** Protein abundance for transcriptional factors of VCAM1 (c-Fos, c-Jun, IRF1, p65, SP1, and GATA6) in the nuclear extracts and the relative quantification data. EA.hy926 cells were pretreated with nuciferine for 2 h, followed by 1 h TNFα incubation. Histone H3 was utilized as the loading control for nuclear proteins. Immunoblots of VCAM1 under 12 h TNFα stimulation in the presence of c-Fos- **(B)** and c-Jun-specific **(C)** siRNAs (si c-Fos and si c-Jun) or scrambled siRNA (positive control). All values are denoted as the means ± SD from at least three independent tests. Each group contains the same amount of solvent. ud: undetectable. ∗*p* < 0.05 and ∗∗*p* < 0.01 indicate statistically significant differences. ns, no significant differences.

Transcriptional activation by nuclear factors, such as activating protein 1 (AP1, the heterodimeric form of c-Fos and c-Jun), nuclear factor-κB (NFκB/p65), GATA-binding protein 6 (GATA6), interferon regulatory factor 1 (IRF1), and specificity protein 1 (SP1), is the main regulation manner for VCAM1 generation (Iademarco et al., 1992; Neish et al., 1992; Neish et al., 1995a; Neish et al., 1995b). As shown in Figure 1C, nuciferine treatment robustly prevented the TNFα-induced transcriptional activity of VCAM1, which spurred us to explore the potential transcriptional regulation mechanism(s) behind nuciferine-reduced VCAM1. We first analyzed the nuclear levels of the aforementioned nuclear factors, and our data showed that TNFα exposure triggered the nuclear expressions of AP1 (c-Fos and c-Jun), IRF1, and GATA6 but not those of NFκB/p65 and SP1, while nuciferine intervention significantly reduced the nuclear levels of c-Fos and c-Jun (components of the heterodimeric AP1) without affecting IRF1 and GATA6 (Figure 6A). Then, to confirm the decisive role of AP1 (c-Fos and c-Jun) in TNFα-induced VCAM1 activation in the present cell setting, specific siRNAs were introduced to knock down c-Fos or c-Jun, respectively. The data revealed that c-Fos or c-Jun was effectively silenced by siRNA (Supplementary Figures S2, S3). Upon genetic knockdown of c-Fos or c-Jun, the TNFα-induced VCAM1 protein was largely abolished (Figures 6B, C). These findings implied that nuciferine alleviated TNFα-induced AP1 activation to prevent VCAM1 activation.

### Autophagy activation contributes to nuciferine-inhibited AP1

**FIGURE 7:**
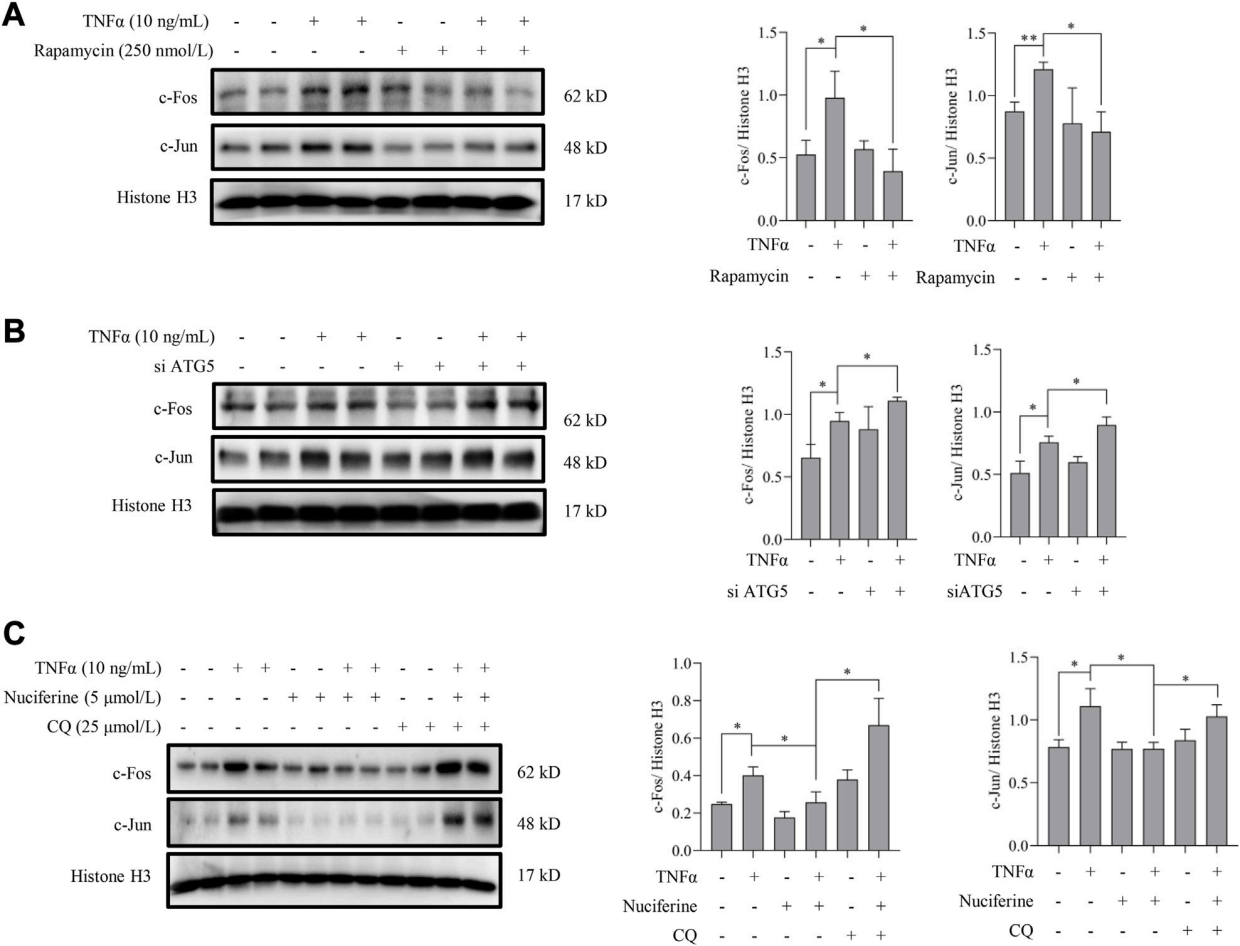
Autophagy activation contributes to nuciferine-inhibited AP1. Immunoblots of nuclear AP1 (c-Fos and c-Jun) and the quantification data in EA.hy926 cells **(A)** pretreated with rapamycin (250 nmol/L) for 2 h or **(B)** pretreated with si ATG5 (or scrambled siRNA) for 24 h, followed by 1 h TNFα incubation. **(C)** Protein abundance and the quantification data of AP1 in the nuclei of EA.hy926 cells pretreated with nuciferine and CQ for 2 h and then incubated with TNFα for another 12 h. Histone H3 was utilized as the loading control for nuclear proteins. Each group contains the same amount of solvent. ∗*p* < 0.05 indicates statistically significant differences.

Next, we investigated whether nuciferine-activated autophagy contributed to its preventive role on AP1. To answer this question, the involvement of autophagy in the regulation of TNFα-induced AP1 activation was first investigated. Rapamycin was introduced to activate autophagy as we described previously, and the nuclear contents of AP1 (c-Fos and c-Jun) were detected in endothelial cells. As shown in Figure 7A, TNFα-induced nuclear translocation of both c-Fos and c-Jun was abolished by rapamycin treatment. In addition, genetically repressing autophagy by knocked down ATG5 enhanced the TNFα-promoted AP1 (c-Fos and c-Jun) level in nucleus (Figure 7B). These results implied that autophagy activation was involved in AP1 inactivation. Furthermore, in the presence of CQ, a special autophagy inhibitor, nuciferine failed to reduce TNFα-induced c-Fos and c-Jun in the nuclei (Figure 7C), which was consistent with the protein expression pattern of VCAM1 under the same treatments (Figure 3D). These results collectively suggested that nuciferine stimulated autophagy to prevent TNFα-induced nuclear AP1 increase and further VCAM1 activation.

## Discussion

**FIGURE 8:**
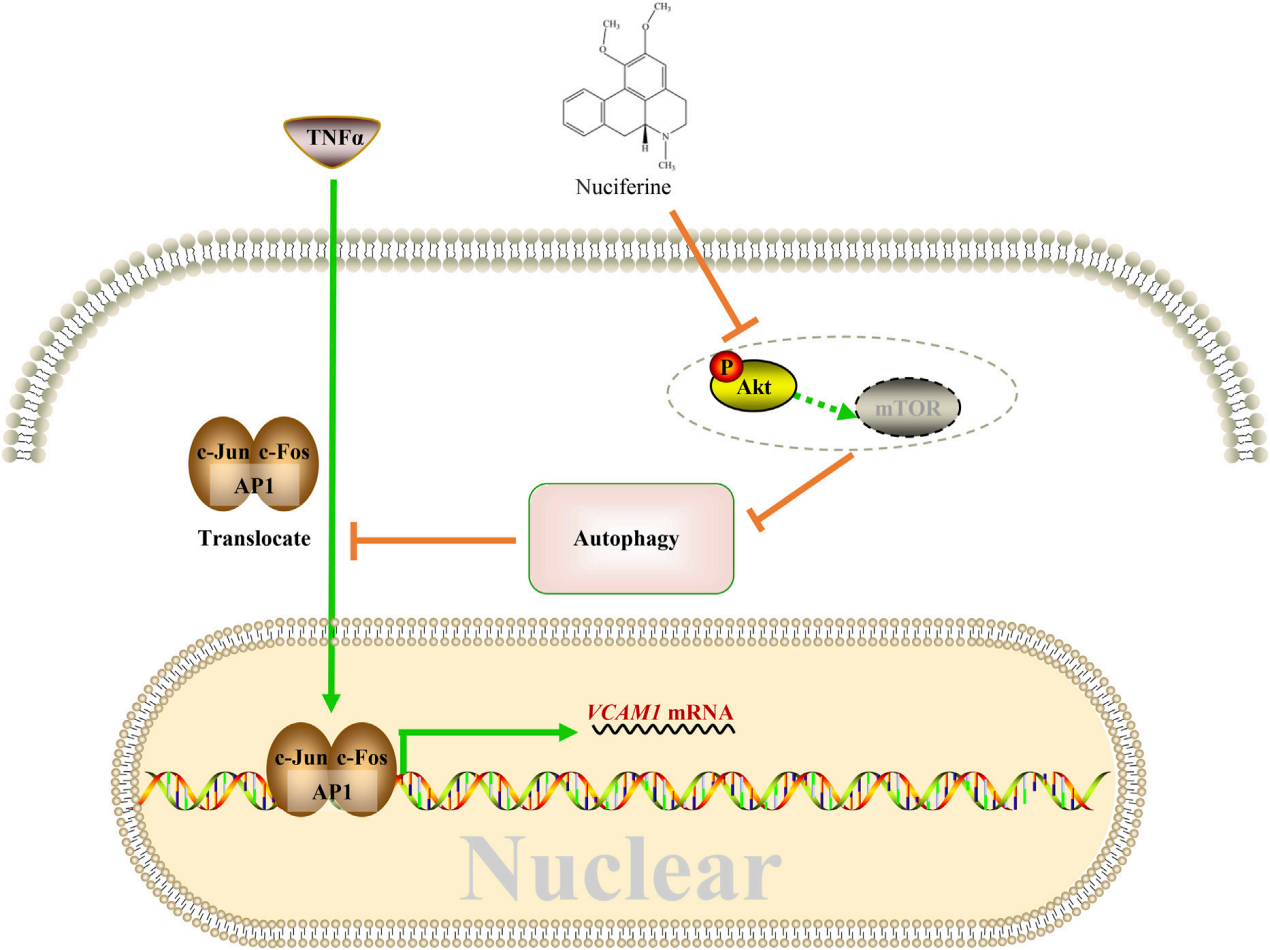
Proposed working model.

This study reported for the first time the protective role of nuciferine against VCAM1 activation in both obesity-associated mice and human vascular endothelial cells. A mechanism study showed that nuciferine activates autophagy via inhibiting the Akt pathway so that in an autophagy-dependent manner, it reduces the nuclear AP1 (specifically refers to the c-Jun and c-Fos components in this study) level, to repress VCAM1 activation through transcriptional regulation ultimately (Figure 8).

The level of VCAM1 is increased (i.e., VCAM1 is activated) in different cardiovascular events and is reported to be positively associated with cardiovascular mortality, clinical improvements, and prognosis in patients (Jager et al., 2000; Blum et al., 2006; Castillo et al., 2009). Thus, blockade of VCAM1 activation is recognized as a potential therapeutic approach to improve cardiovascular events. VCAM1 has been reported to be blocked through various approaches, such as neutralizing antibody, genetical modification, or chemical compounds (*e.g.,* AGI-1067), to improve atherosclerosis, rheumatoid arthritis (RA), nonalcoholic steatohepatitis (NASH), and so on (Carter et al., 2002; Park et al., 2013; Furuta et al., 2021). Furthermore, in this study, nuciferine was newly reported as the phytogenic compound to block TNFα-induced VCAM1 activation.

In the following mechanistical studies, we found that nuciferine could increase the protein level of autophagy initiation-related proteins (Beclin1, ATG5, and ATG12) and the number of autophagy puncta, indicating its autophagy-inductive capacity in vascular endothelial cells. This is consistent with the previous study conducted by Shi *et al.* which showed that nuciferine induces the expression of ATG5 protein, as well as promotes the formation of autophagic puncta and autophagosomes in Caco-2 and HT-29 cell lines (human colonic epithelial tumor cell lines isolated from colorectal adenocarcinoma) (Shi et al., 2021). Since autophagy induction has been considered a therapeutically viable approach for cardiovascular events (Lavandero et al., 2015), compounds that induce autophagy have been investigated for the intervention of these diseases. For example, clonidine and rilmenidine (inducing autophagy through the reduction in cAMP levels) are approved for the clinical treatment of hypertension (Leidal et al., 2018); rapamycin (inducing autophagy through mTOR inhibition) exerted anti-hypertrophic and anti-inflammatory efficiency in the aged heart in an animal study (Flynn et al., 2013). Herein, nuciferine was finally demonstrated to prevent VCAM1 activation through autophagy stimulation in the endothelial cells.

Then, we attempted to investigate how nuciferine stimulated autophagy. AMPK and SIRT1 pathways are generally accepted upstream signals involved in autophagy stimulation. Referring to previous studies, AMPK activates autophagy in the initiation stage through mTOR complex 1 (mTORC1) inhibition, mammalian autophagy-initiating kinase ULK1 activation through phosphorylation, or Beclin1-VPS34-VPS15 complex activation in the presence of ATG14L (Egan et al., 2011; Kim et al., 2011); SIRT1 mainly activates autophagy via its deacetylase activity to induce the expression of ATGs or fork-head box protein O1 (FOXO1) (Lee et al., 2008; Hariharan et al., 2010). However, nuciferine had no influences on these two molecules under our experimental setting in the human vascular endothelial cells, whereas p38 MAPK signaling was significantly repressed upon nuciferine treatment. p38 MAPK signaling is mainly activated by inflammatory and cellular stress stimuli and involves the negative control of autophagy under different cell settings (Webber and Tooze, 2010). SB202190, an antagonist of the p38 MAPK pathway specifically targeting p38α/β, is demonstrated as an autophagy inducer through p38 inhibition (Comes et al., 2007). Although SB202190 activated autophagy and prevented TNFα-induced VCAM1 activation in our experimental setting as well (Figures 4B, C), activating the p38 MAPK pathway by asiatic acid had no influence on the protective role of nuciferine against TNFα-induced VCAM1 activation (Figure 4D). In fact, it has been proposed that pyridinyl imidazole class inhibitors, such as SB202190, alter autophagy flux and autophagy-promoting gene expression in a cell type-specific, MAPK14/p38α-MAPK11/p38β-independent manner (Menon et al., 2015). Thus, p38 MAPK is irrelevant to autophagy activation and VCAM1 reduction in response to nuciferine.

As nuciferine was found to induce the protein level of Beclin1, ATG12, and ATG5 (Figure 3C), we further verified the involvement of mTOR-related pathways, which governs autophagy and lysosomal biogenesis (Napolitano and Ballabio, 2016). Akt signaling is one of the mTOR-related pathways and could regulate autophagy activity via mTOR. In the current study, nuciferine treatment led to the dephosphorylation of both Akt (Ser473) and p70S6K (Thr389, one of the downstream targets of mTOR), implying a negative regulation of the Akt/mTOR pathway by nuciferine (Figure 5B). As a coincidence to our finding on Akt, nuciferine has been recently reported to block the activation of the PI3K/Akt signaling pathway via calmodulin 4 to suppress the proliferation and migration of the vascular smooth muscle cells as a potential drug against atherosclerosis (Xiao et al., 2023). Thus, we further investigated whether nuciferine stimulated autophagy through the Akt/mTOR pathway. Our data showed that similar to nuciferine, suppressing Akt by the specific antagonist MK-2206 stimulated autophagy and protected human vascular endothelial cells from TNFα-induced VCAM1 activation. In addition, activating Akt with insulin repressed autophagy and diminished protection against TNFα-induced VCAM1 activation induced by nuciferine. Collectively, nuciferine was proved to stimulate autophagy to protect VCAM1 activation through the Akt/mTOR pathway.

Next, the autophagy-dependent protective role of nuciferine against TNFα-induced VCAM1 was elucidated. VCAM1 is rarely expressed under physical conditions but could be quickly activated by a pro-inflammatory factor, including TNFα, which regulates the activation, maturation, and cytokine and chemokine release of leukocytes, thus playing a vital and direct role in endothelial dysfunction (Zhang et al., 2009). Upon stimulation, VCAM1 expression is transcriptionally activated by its transcription factors (Furuta et al., 2021), including AP1, NF-κB (p65), IRF1, GATAs, and SP1(Iademarco et al., 1992; Neish et al., 1992; Neish et al., 1995a; Neish et al., 1995b). In this study, TNFα was found to stimulate the nuclear translocation to activate these transcription factors except for SP1, which might be due to the experiment settings. Among these transcription factors, AP1 is a cluster of heterodimeric transcription factors, which could be activated by various stimuli, including inflammatory cytokines (Wisdom, 1999). c-Jun and c-Fos were picked up to represent AP1 since c-Jun is the most potent subunit among AP1 members and forms a stable heterodimer with c-Fos (Shaulian and Karin, 2002). Furthermore, for the first time, this study proved that nuciferine treatment alleviates TNFα-induced AP1 activation, which contributes to its protection against VCAM1 activation, but the mechanism remains to be elucidated.

## Data Availability

The original contributions presented in the study are included in the article/Supplementary Material; further inquiries can be directed to the corresponding author.
