# Supplementary figures and images for: Nuciferine induces autophagy to relieve vascular cell adhesion molecule 1 activation via repressing the Akt/mTOR/AP1 signal pathway in the vascular endothelium
Source: Front Pharmacol. 2023 Sep 28;14:1264324. doi: 10.3389/fphar.2023.1264324 (PMC10569124; doi:10.3389/fphar.2023.1264324)

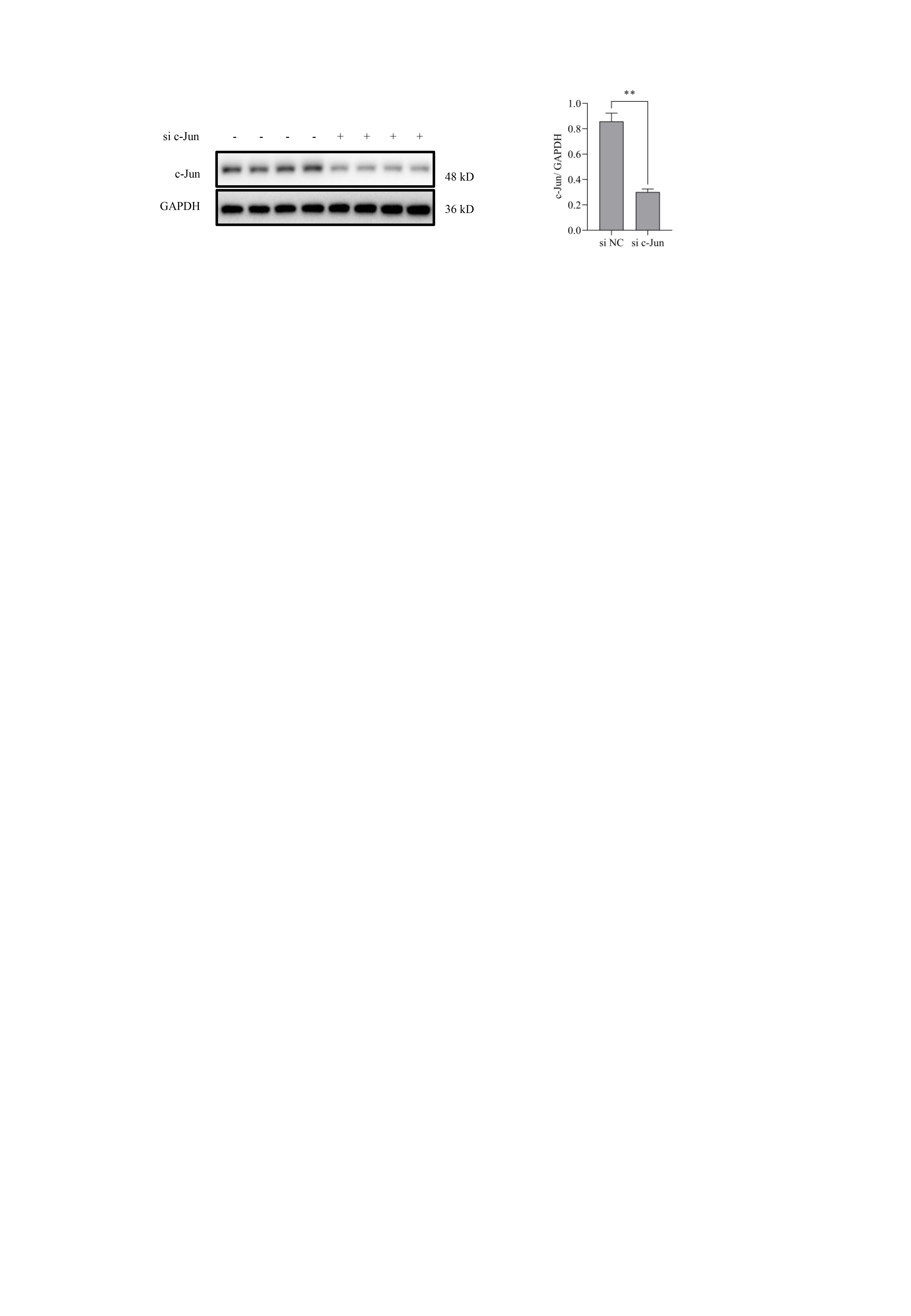

Supplement: Supplementary file 1 [file Image3.tif]

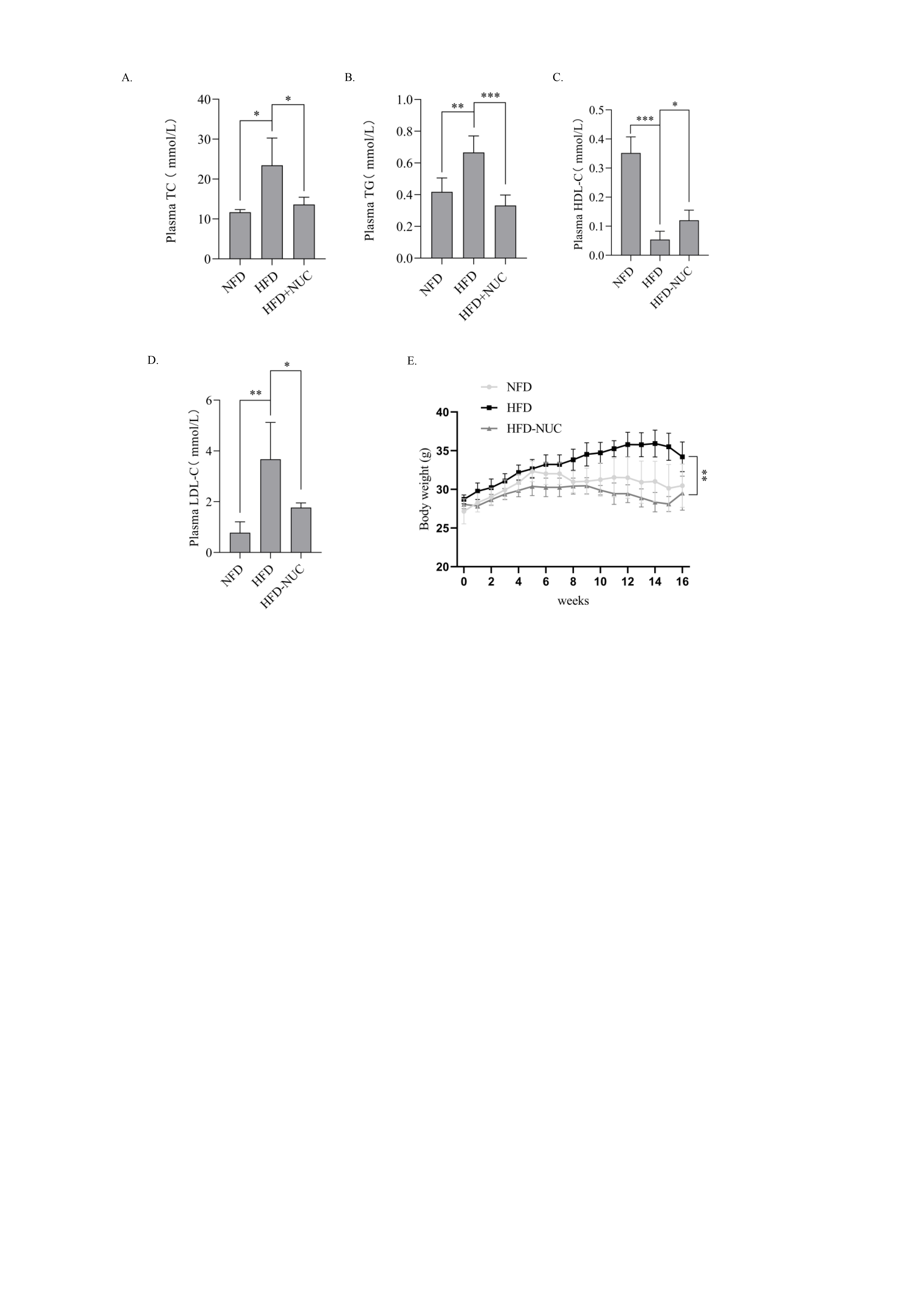

Supplement: Supplementary file 2 [file Image4.tif]

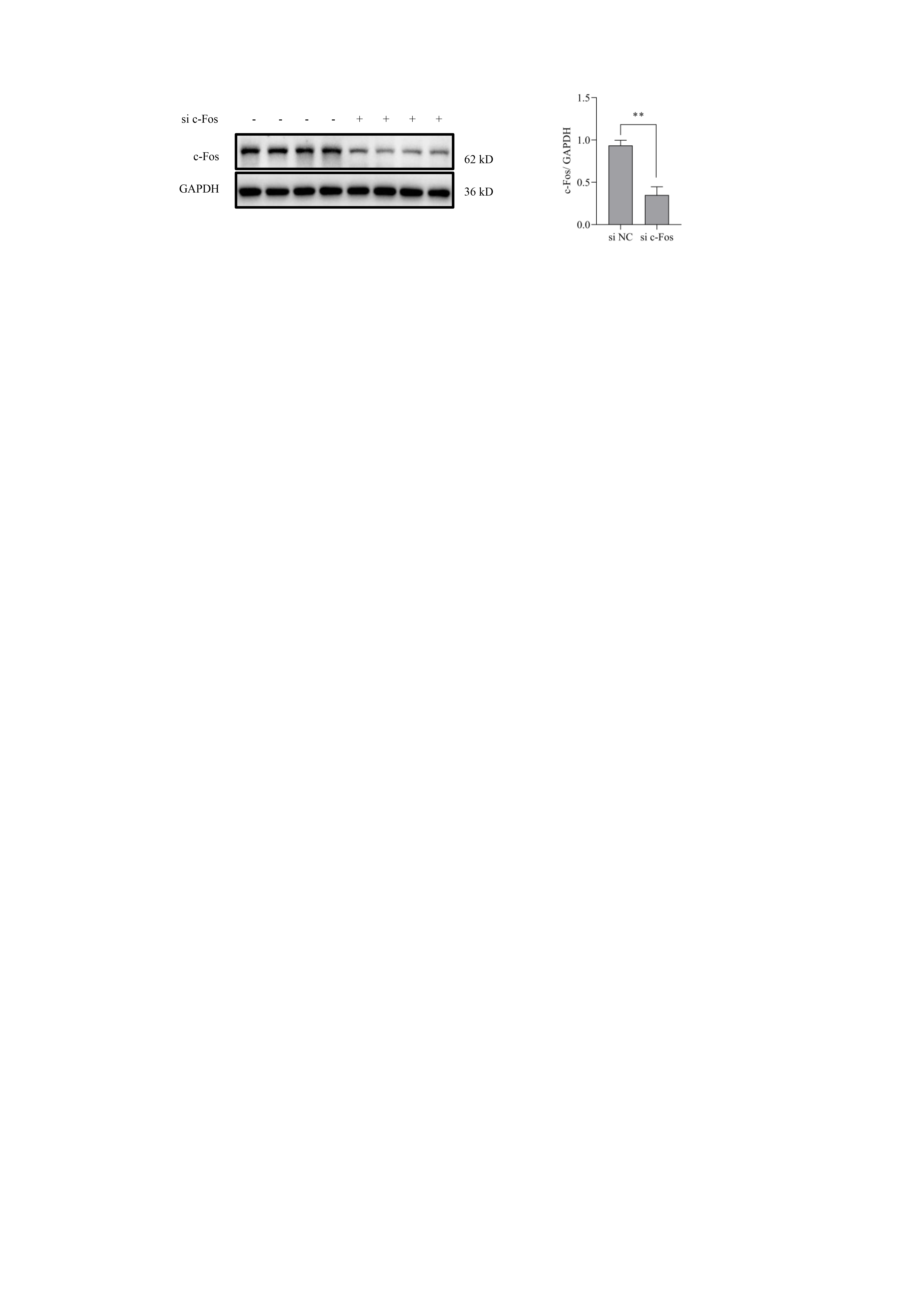

Supplement: Supplementary file 3 [file Image2.tif]

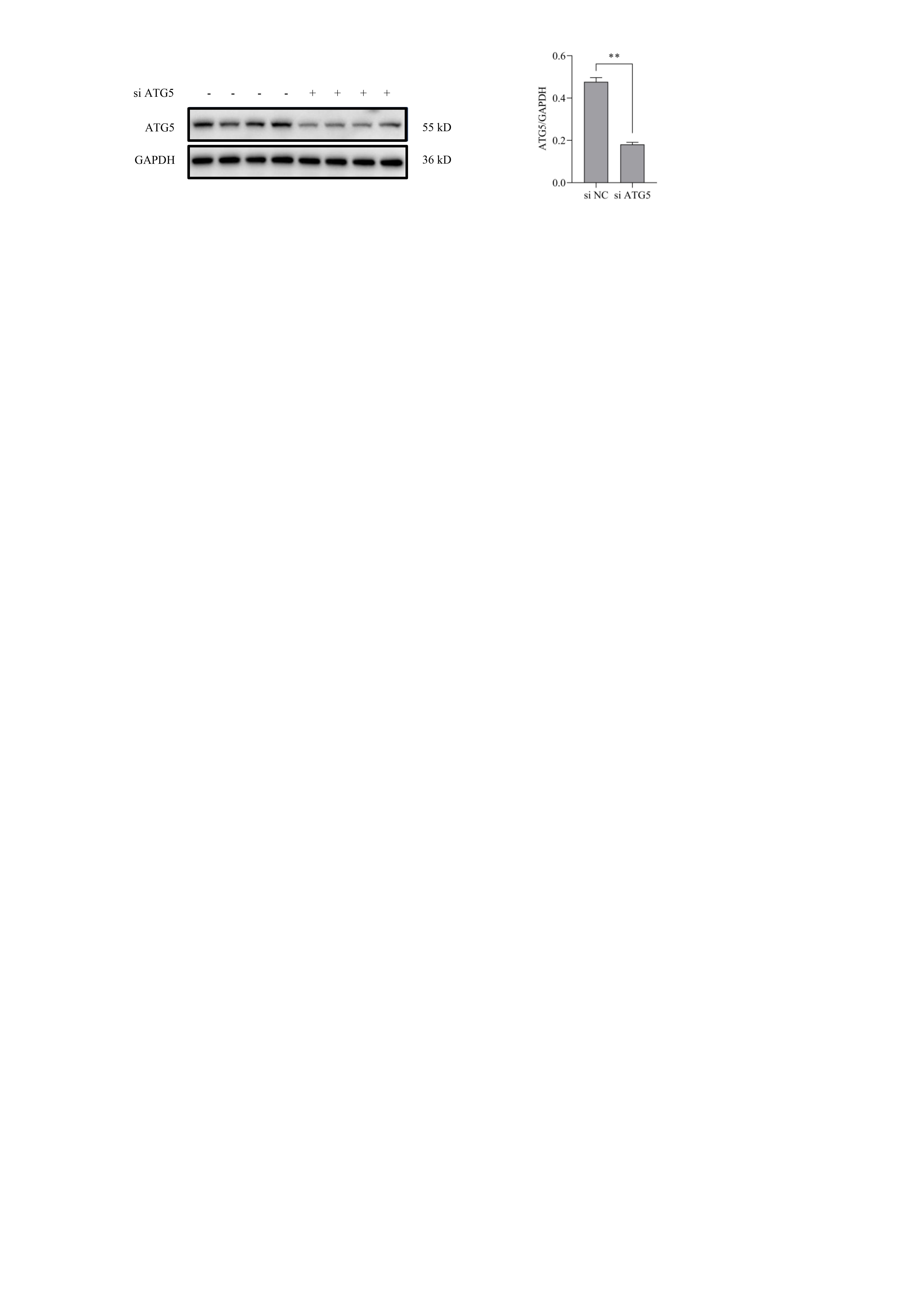

Supplement: Supplementary file 4 [file Image1.tif]
