# Supplementary material for: Nuciferine induces autophagy to relieve vascular cell adhesion molecule 1 activation via repressing the Akt/mTOR/AP1 signal pathway in the vascular endothelium
Source: Front Pharmacol. 2023 Sep 28;14:1264324. doi: 10.3389/fphar.2023.1264324 (PMC10569124; doi:10.3389/fphar.2023.1264324)
Supplement: Supplementary file 5 [file DataSheet1.docx]

Supplementary Material

## Supplementary Figures

Figure S1. Knockdown efficiency of ATG5 protein and the quantification data. All values are denoted as means ± SD from at least three independent tests. ∗*P* < 0.05 indicate statistically significant differences.

Figure S2. Knockdown efficiency of c-Fos and the quantification data. All values are denoted as means ± SD from at least three independent tests. ∗*P* < 0.05 indicate statistically significant differences.

Figure S3. Knockdown efficiency of c-Jun and the quantification data. All values are denoted as means ± SD from at least three independent tests. ∗*P* < 0.05 indicate statistically significant differences.

Figure S4. The biochemical indicators and body weight in mice. **(A)** Plasma TC level in mice. **(B)** Plasma TG level in mice. **(C)** Plasma HDL-C level in mice. **(D)** Plasma LDL-C level in mice. **(E)** The body weight of the mice. All values are denoted as means ± SD from five animal samples. ∗*P* < 0.05, ∗∗*P* < 0.01 and ∗∗∗*P* < 0.001 indicate statistically significant differences. TC, total cholesterol; TG, triglyceride; HDL-C, high density lipoprotein cholesterol; LDL-C, low density lipoprotein cholesterol.
